# Fulminant Immune Checkpoint Inhibitor-Induced Myocarditis and Complete Heart Block in Advanced Melanoma: A Case Report

**DOI:** 10.7759/cureus.98075

**Published:** 2025-11-29

**Authors:** Bakr Alhayek, Xiaowei Malone, Muhammad Affan Rashid, Lidia Sepulveda, Asha Ramsakal

**Affiliations:** 1 Internal Medicine, AdventHealth Tampa, Tampa, USA

**Keywords:** autoimmune cardiotoxicity, complete heart block, fulminant myocarditis, immune checkpoint inhibitors (icis), immune-related adverse events (iraes)

## Abstract

Immune checkpoint inhibitor-associated myocarditis is an uncommon, yet serious, complication. We describe a 75‑year‑old man with stage IIIB NRAS‑mutant melanoma who received neoadjuvant ipilimumab, nivolumab, and relatlimab. Within days, he developed fever, diffuse rash, and myalgias; laboratory evaluation revealed a creatine kinase of 1,875 U/L and a high‑sensitivity troponin I level of approximately 2,700 ng/L. A 12‑lead electrocardiogram showed new right bundle branch block and anterior T‑wave inversions, prompting suspicion of immune‑mediated myocarditis. He was treated with pulse‑dose methylprednisolone and mycophenolate; however, troponin levels continued to rise (>12,000 ng/L), and conduction disease progressed to complete atrioventricular block requiring emergent transvenous pacing. Despite the continuation of high‑dose corticosteroids and the addition of abatacept for presumed steroid‑refractory disease, he developed a sustained monomorphic wide‑complex tachycardia approximately four days after transfer and died despite cardioversion.

This case underscores the malignant arrhythmic phenotype of fulminant immune‑checkpoint myocarditis and highlights the need for rapid recognition, immediate initiation of high‑dose immunosuppression, and early escalation to second‑line therapies when electrical instability persists.

## Introduction

Immune checkpoint inhibition has revolutionized oncologic care by unleashing cytotoxic T‑lymphocyte activity against tumor cells [1]. In metastatic melanoma, dual CTLA‑4/PD‑1 blockade with ipilimumab plus nivolumab confers superior response and overall survival compared with monotherapy. Nevertheless, the same immune disinhibition underlies a distinctive spectrum of immune‑related adverse events (irAEs) [2]. Although the reported incidence of immune checkpoint inhibitor (ICI)‑mediated myocarditis ranges from 0.06% to 1%, it carries a disproportionate mortality, approaching 40% [3,4].

Myocardial inflammation may involve both the working myocardium and specialized conduction tissues, manifesting as heart failure, cardiogenic shock, and malignant arrhythmias [5]. Pathology studies demonstrate dense CD4⁺/CD8⁺ lymphocytic infiltration with myocyte necrosis, and, in up to one‑third of fatal cases, concurrent skeletal‑muscle or neuromuscular involvement that portends poorer outcomes [6,7]. Risk is highest with combination ICI therapy, pre‑existing cardiovascular disease, older age, and early cycles of treatment [2,3,6,7]. Prompt recognition and immunosuppression are therefore paramount. In this report, we present a case of ICI‑induced myocarditis in metastatic melanoma, highlighting the challenges of management.

## Case presentation

A 75‑year‑old man with Grover disease, hypertension, obesity, hyperlipidemia, and a remote stage III cutaneous melanoma of the posterior trunk (>20 years earlier; wide local excision, positive sentinel node, completion cervical lymphadenectomies, and adjuvant interferon therapy) later developed multiple additional melanocytic lesions. These included melanoma in situ of the left neck (positive margins and re‑excised) and an atypical melanocytic lesion of the left thigh (positive margins and no invasion). More recently, he was diagnosed with an NRAS‑mutant invasive melanoma of the right lateral neck, pT2b (AJCC: Breslow >2-4 mm with ulceration), treated by radical wide local excision after unsuccessful sentinel lymph node mapping [8]. Staging positron emission tomography/computed tomography (PET/CT) and ultrasound demonstrated biopsy‑proven metastasis to a right cervical lymph node without distant disease; overall clinical stage IIIB (N1b and M0) [8]. He enrolled in a neoadjuvant trial and received cycle 1, day 1, triple ICI therapy with ipilimumab, nivolumab, and relatlimab.

Within approximately three days of treatment, he developed fever (~101°F), heart rate 96, respiratory rate 22, blood pressure 145/75, rigors, and a severe diffuse erythematous eruption far worse than his baseline Grover disease. Topical triamcinolone and an oral methylprednisolone taper were ineffective. Over subsequent days, he developed escalating systemic symptoms, severe headaches, generalized myalgias, and profound fatigue. He was admitted to the referring cancer center for evaluation of persistent headache, fever, diffuse rash, myalgias, and weakness. Initial laboratories are summarized in Table 1; brain CT and magnetic resonance imaging (MRI) showed no acute intracranial process.

**Table 1:** Baseline Lab Values

Lab Test	Result	Reference Range
Sodium	137 mmol/L	135-145 mmol/L
Potassium	4.4 mmol/L	3.5-5.1 mmol/L
Chloride	106 mmol/L	98-107 mmol/L
Glucose	81 mg/dL	70-99 mg/dL (fasting)
Blood Urea Nitrogen (BUN)	11 mg/dL	7-20 mg/dL
Creatinine	1.0 mg/dL	0.6-1.3 mg/dL
Calcium	9.1 mg/dL	8.5-10.5 mg/dL
Phosphorus	3.8 mg/dL	2.5-4.5 mg/dL
Albumin	3.8 g/dL	3.4-5.0 g/dL
Total Bilirubin	0.5 mg/dL	0.1-1.2 mg/dL
Alkaline Phosphatase	55 U/L	40-130 U/L
Aspartate Aminotransferase (Serum Glutamic-Oxaloacetic Transaminase), or AST (SGOT)	92 U/L	10-40 U/L
Alanine Aminotransferase (Serum Glutamic Pyruvic Transaminase), or ALT (SGPT)	58 U/L	7-56 U/L
Creatine Kinase (CK)	1875 U/L	22-198 U/L
Magnesium	2.2 mg/dL	1.6-2.6 mg/dL
Lactic Acid	1.5 mmol/L	0.5-2.0 mmol/L
Procalcitonin	0.2 ng/mL	<0.1 ng/mL
White Blood Cell	5.29 k/uL	4.0-10.0 k/uL
Red Blood Cell	4.59 mil/uL	4.2-5.9 mil/uL
Hemoglobin	14.4 g/dL	13.5-17.5 g/dL
Platelet Count	205 k/uL	150-400 k/uL

Ongoing myalgias and rising CK levels prompted expanded immune‑toxicity screening. High‑sensitivity troponin I was detected at ~2,700 ng/L (upper limit of normal, <20 ng/L), despite the absence of cardiac complaints, prompting an urgent cardiology consultation. A 12‑lead electrocardiogram (ECG) showed sinus rhythm at approximately 60 beats min⁻¹, with new right bundle branch block and anterior T‑wave inversions in V₁-V₂; mild lateral T‑wave changes in I/aVL were unchanged from a pretreatment staging ECG (baseline image not retrievable). Transthoracic echocardiography demonstrated normal biventricular size and systolic function (left‑ventricular ejection fraction (LVEF), 65%-70%) without regional wall‑motion abnormality or pericardial effusion. In the context of recent triple ICI exposure, marked troponinemia, elevated CK, new conduction abnormality, and multisystem symptoms, probable ICI myocarditis with possible concurrent ICI myositis (≥ grade 2) was diagnosed jointly by oncology and cardiology.

Management was escalated to pulse intravenous methylprednisolone 1 g/day for three days, followed by a planned 1 mg/kg/day taper; mycophenolate mofetil (MMF) 1 g twice daily was added for a suspected multi‑organ irAE. Serial troponin values continued to rise (~3.7 k → ~5.5 k → ~7.6 k ng/L) and ultimately exceeded 12 k ng/L before transfer; CK also climbed >2.4 k U/L, and B‑type natriuretic peptide was approximately 140 pg/mL. Over the next 48-72 hours, he developed intermittent high‑grade atrioventricular (AV) block, evolving on the right bundle branch background, with episodes of Mobitz II block, transient complete heart block, and brief asystolic pauses accompanied by presyncope and short convulsive spells. Multiple doses of atropine were administered for bradycardia. Vasoactive support (dobutamine, followed by dopamine; epinephrine was considered when isoproterenol was unavailable) was used as blood pressure fluctuated from 140 mmHg to approximately 90 mmHg systolic. Sedation with dexmedetomidine was discontinued because of bradycardia. An arterial line was placed for invasive hemodynamic monitoring. Point‑of‑care cardiac ultrasound continued to show preserved left‑ventricular function, although right‑ventricular wall motion was difficult to assess. Given escalating conduction instability and anticipated pacing requirements, transfer to our tertiary cardiovascular intensive‑care unit was arranged.

**Figure 1:**
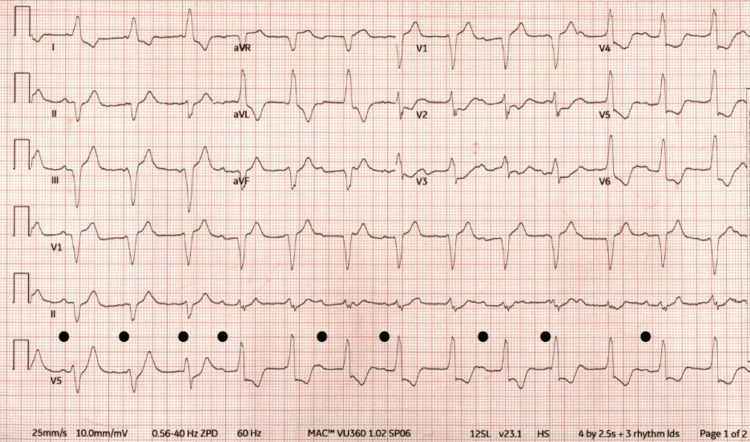
Complete Heart Block With Infranodal Escape in ICI Myocarditis A 12‑lead ECG, on transfer to the receiving center, shows complete AV block with non‑conducted sinus P waves (black dots) and a slow junctional or ventricular escape rhythm at approximately 45 beats/min. QRS complexes are wide (~160 ms), in a right bundle branch block/left anterior fascicular block pattern, with secondary ST‑T changes. The ECG was recorded at a paper speed of 25 mm/s and a gain of 10 mm/mV [9]. ECG: electrocardiogram; AV: atrioventricular; ICI: immune checkpoint inhibitor

Upon arrival, he exhibited complete AV block on a background of right bundle branch block with a slow junctional escape rhythm (Figure 1). A transvenous pacemaker was inserted emergently (backup rate 60 beats/min). The pulse‑dose steroid course was completed, and he was transitioned to a high‑dose methylprednisolone taper (1 mg/kg/day equivalent) with continuation of MMF. Due to persistent biomarker elevation and electrical instability, abatacept was added as second‑line immunomodulatory therapy. Serial biomarkers remained profoundly abnormal (high‑sensitivity troponin I >10 k ng/L).

**Figure 2:**
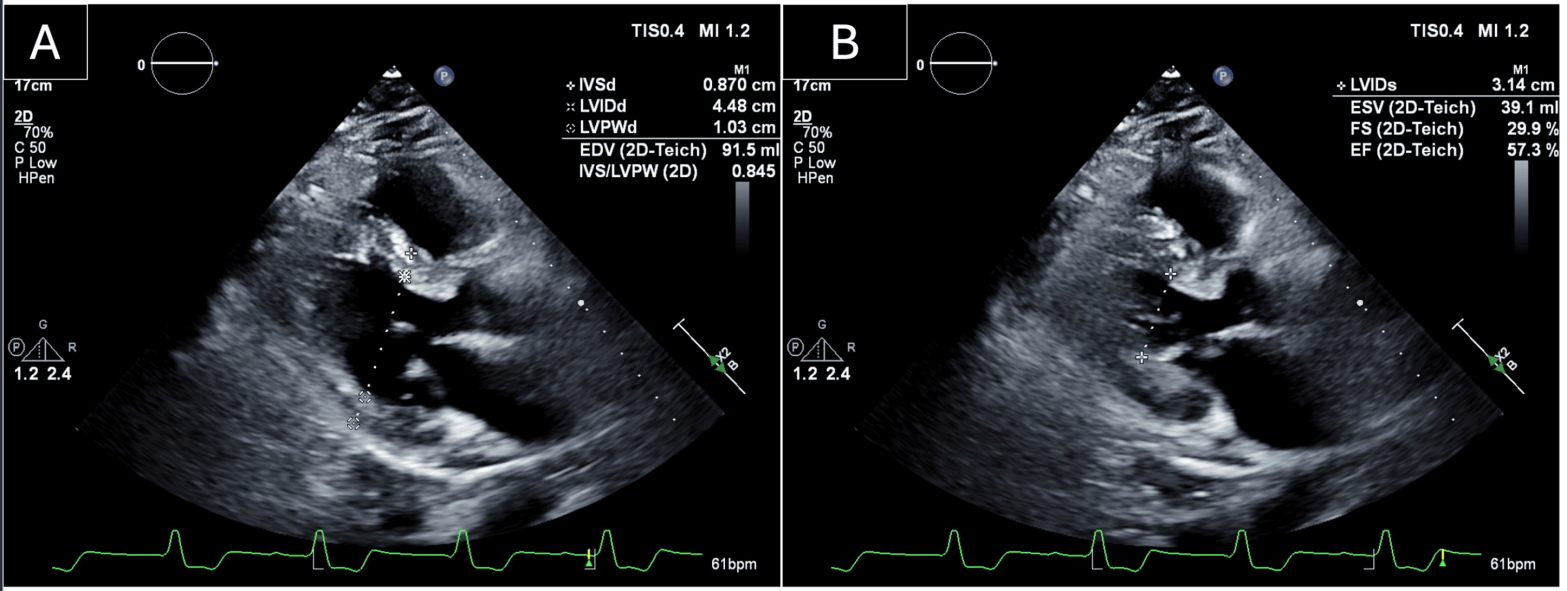
Echocardiographic Views in Immune Checkpoint Inhibitor-Associated Myocarditis (A) Parasternal long‑axis view at end‑diastole, with calipers measuring the left‑ventricular internal diameter in diastole (LVIDd = 4.48 cm); measurement points are shown. (B) Parasternal long‑axis view at end‑systole, demonstrating LVIDs = 3.14 cm; fractional shortening (29.9%) and end‑systolic volume (39.1 mL) were calculated using the Teichholz linear method [10].

**Figure 3:**
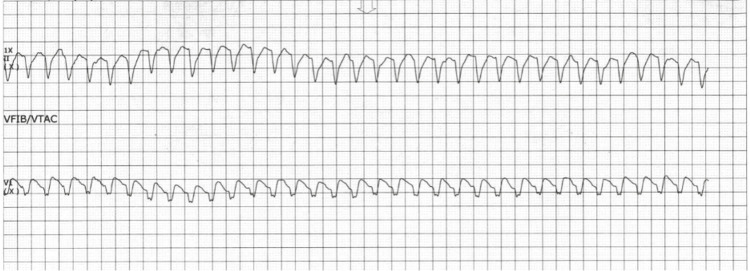
Sustained Wide-Complex Tachyarrhythmia Consistent With Ventricular Tachycardia in ICI Myocarditis Telemetry strip (lead II, 25 mm/s, 10 mm/mV) shows a regular, wide‑complex tachycardia at approximately 200 beats/min, without discernible atrial activity. White arrows indicate the onset of tachycardia and representative QRS complexes. Intravenous adenosine (6 mg, followed by 12 mg) produced no slowing; synchronized cardioversion (150 J) failed, and the rhythm progressed to pulseless electrical activity. ICI: immune checkpoint inhibitor

Repeat comprehensive echocardiography now demonstrates new, mild, global LV systolic dysfunction (LVEF 45%-50%), consistent with progressive inflammatory myocardial injury (Figure 2). Bedside telemetry (lead II at the top, precordial monitoring lead at the bottom), obtained approximately four days after transfer, showed a rapid, regular, monomorphic, wide‑complex tachycardia (~200 beats/min) (Figure 3). No organized atrial activity or response to adenosine was observed; 6 mg, then 12 mg, of adenosine produced no effect. Endotracheal intubation and synchronized cardioversion (150 J) were performed. Despite these measures, he progressed to pulseless electrical activity and died shortly thereafter. Per previously expressed wishes, resuscitative efforts were discontinued in agreement with his family. No autopsy was performed.

He subsequently progressed to pulseless cardiovascular collapse. Despite advanced resuscitation efforts, a sustained return of spontaneous circulation was not achieved. Per previously expressed wishes, resuscitative efforts were discontinued in agreement with the family. He died shortly thereafter, demonstrating the fulminant, arrhythmogenic phenotype of severe ICI‑associated myocarditis, despite guideline‑directed, multi‑agent immunosuppression (high‑dose corticosteroids, MMF, abatacept) and temporary pacing support. No autopsy was performed.

## Discussion

ICIs have redefined the treatment landscape for advanced and metastatic melanoma, offering substantial survival benefits compared with traditional cytotoxic chemotherapy regimens [11]. However, along with these advances, comes a spectrum of irAEs that can affect virtually any organ system [12]. Cardiotoxicity, although relatively rare, poses a uniquely severe and potentially fatal threat due to immune‑mediated myocardial inflammation and dysfunction [12,13]. ICI‑associated myocarditis is notable for its fulminant course and high mortality [14]. Excessive T‑cell activation and infiltration into the myocardium and conduction system result in direct myocyte damage, edema, and fibrosis [13].

Cardiac arrhythmias encompass a broad spectrum of rhythm disturbances, ranging from incidental premature beats to malignant tachyarrhythmias or high‑grade heart block, which can acutely compromise cardiac output [15]. Severe arrhythmias, whether bradyarrhythmias such as advanced AV block, or rapid ventricular tachyarrhythmias, may precipitate hypotension, syncope, or sudden cardiac death if not promptly managed [15]. In the context of ICI‑induced myocarditis, arrhythmias represent a particularly ominous manifestation of fulminant cardiac inflammation. Emerging data suggest that ICI myocarditis is highly arrhythmogenic, with one literature review reporting complete AV block in about 25% of cases, and ventricular tachycardia in ~13% [16]. These rhythm disturbances often herald a malignant course; in a multicenter registry of ICI myocarditis, nearly half of patients experienced major adverse cardiac events, despite immunosuppressive therapy [17]. Conduction disturbances, such as AV block, can be especially sinister, reflecting inflammation of the conduction tissue [18]. As illustrated by our case, the abrupt onset of complete AV block, accompanied by malignant ventricular arrhythmias, can quickly progress to cardiogenic shock and death despite aggressive management.

Arrhythmia burden in ICI myocarditis exceeds that seen in other inflammatory cardiomyopathies [19]. In the International ICI‑Myocarditis Registry, life‑threatening ventricular arrhythmias occurred more often than in grade 2R/3R cardiac allograft rejection; the development of complete heart block, or malignant ventricular arrhythmia, independently predicted myocarditis‑related mortality [19,20]. Subsequent multicenter data confirmed that complete heart block is a strong, adverse prognostic marker in ICI myocarditis [19,20]. Systematic ECG reviews further show that new bundle‑branch block, low QRS voltage, pathologic Q waves, and diverse supraventricular or ventricular tachyarrhythmias are common electrical signatures in affected patients [21,22].

While clinicians conventionally rely on echocardiography, ECG changes, and myocardial biomarkers (e.g., troponin) to evaluate for myocarditis, these tools may be nondiagnostic in early or mild disease [23,24]. For instance, a normal ejection fraction does not rule out incipient myocarditis [23,24]. More sensitive modalities, such as cardiac magnetic resonance imaging (CMR), may detect myocardial edema or fibrosis, but can be impractical in hemodynamically unstable patients [25]. In any ICI‑treated patient presenting with new conduction abnormalities, elevated cardiac enzymes, or hemodynamic compromise, a high index of suspicion and early involvement of cardio‑oncology services are imperative.

Certain factors may predispose patients to higher risks of ICI‑induced myocarditis, including older age, pre‑existing cardiovascular disease, and the use of combination ICI regimens [26]. Clinical manifestations range from asymptomatic biomarker elevation to overt heart failure, fulminant arrhythmias, or refractory cardiogenic shock [18]. Management centers on two imperatives: immediate, aggressive immunosuppression and proactive rhythm support. Observational data suggest benefit from pulse‑dose methylprednisolone, followed by ≥1 mg/kg/day taper, with escalation to agents such as mycophenolate and abatacept in steroid‑refractory cases [27,28]. Because conduction disturbances may progress rapidly, continuous telemetry, with a low threshold for temporary pacing, is advised; emerging cardio‑oncology experience supports active‑fixation temporary systems and individualized decisions about permanent pacing once inflammation subsides [22]. Targeted T‑cell costimulation blockade with abatacept, alone or in combination strategies (e.g., ruxolitinib for severe cardio‑muscular overlap), has shown promise in uncontrolled series and is the subject of ongoing prospective evaluation [29-31]. Nonetheless, as shown in this case, even aggressive immunosuppression and mechanical support can fail to halt the rapid progression to lethal arrhythmias and cardiac arrest.

Improved risk‑stratification tools, biomarker development, and standardized monitoring guidelines may help identify high‑risk individuals before fulminant myocarditis develops. Additional research is needed to delineate the most effective immunomodulatory regimens for ICI‑induced cardiotoxicity, and to investigate strategies that preserve antitumor efficacy while reducing off‑target cardiac effects.

## Conclusions

In summary, this case highlights the critical need for heightened vigilance, rapid recognition, and aggressive management of ICI‑related myocarditis, particularly when severe conduction disturbances arise. Despite the significant survival benefits conferred by immune checkpoint blockade, clinicians must remain cognizant of the minor, yet serious, risk of life‑threatening cardiotoxicity. Early, collaborative, multidisciplinary intervention offers the greatest likelihood of preventing tragic outcomes in this exponentially increasing era of immuno‑oncology.
